# Leiomyomatosis peritonealis disseminata associated with endometriosis: A case report and review of the literature

**DOI:** 10.3892/ol.2014.2741

**Published:** 2014-11-27

**Authors:** RULIN YANG, TIANMIN XU, YINGWEI FU, SONGHUA CUI, SHULI YANG, MANHUA CUI

**Affiliations:** Department of Gynaecology and Obstetrics, The Second Hospital of Jilin University, Changchun, Jilin 130041, P.R. China

**Keywords:** leiomyomatosis, peritonealis disseminata, laparoscope, hysteromyomectomy, uterine fibroid, laparoscopic power morcellators

## Abstract

Leiomyomatosis peritonealis disseminata (LPD) is a specific type of leiomyomatosis with an unclear pathogenesis that is rarely diagnosed by clinical evaluation. To date, <200 cases have been reported. The majority of the patients have a medical history of laparoscopic myomectomy for uterine fibroids. The use of laparoscopic power morcellation may be a contributor to the development of LPD, therefore, the specific surgical approach used in laparoscopic myomectomy should be carefully considered, and protective measures should be taken to prevent myoma fragments spreading if laparoscopic power morcellation is used. The present study reviewed and analyzed the medical history, diagnostic process and treatment strategy of a case of LPD to improve our understanding of the disease. In this report, the case of a 34 year-old female who underwent laparoscopic myomectomy to remove a uterine fibroid is presented. During the surgery, a myoma was resected using morcellators. Three years after surgery, exploratory laparotomy was performed due to uterine fibroid recurrence. During surgery, myoma was identified at the uterine bladder peritoneal reflection, where several unequally sized leiomyoma tubercles were identified on the uterine surface. Subsequently, myomectomy was performed. Postoperative pathology diagnosed leiomyoma. Two years later, gynecological ultrasound revealed a mass in the abdomen. Exploratory laparotomy was subsequently performed. During surgery, compact myoma tubercle-like cysts were identified on the surface of the intestine and mesentery, and an endometriotic cyst was identified on the left ovary. As the myomas were too compact to remove completely, the majority of leiomyoma on the intestine and mesentery was resected. The endometriotic cyst on the left ovary was also resected. Considering the patient’s medical history, observations during surgery and pathological results, the final diagnosis was LPD. Following surgery, the patient was treated with the gonadotropin-releasing hormone agonist, triptorelin acetate (3.5 mg, once every four weeks), for three months and followed-up every six months. In October 2014, a gynecological sonography examination revealed no abnormalities and at the time of writing, the patient remains alive and well.

## Introduction

Leiomyomatosis peritonealis disseminata (LPD) is a specific type leiomyomatosis that is rarely identified by clinical evaluation. To date, <200 cases have been reported in the literature. Accurate diagnosis is difficult prior to surgery, as diagnosis relies on medical history, observations during surgery and pathological results. At present, no standard treatment for LPD has been identified. The prognosis of LPD is good as only 9 of ~200 cases that has been reported so far have been malignant (1–8). It has been reported that the majority of LPD patients have previously been exposed to laparoscopic power morcellation during hysterectomy or myomectomy for uterine fibroids (9). The Food and Drug Administration (FDA) (10) have indicated that the use of laparoscopic power morcellation may be associated with the development of LPD. The present study analyzes the clinical procedure and treatment strategy received by an LPD patient who was treated in the Second Hospital of Jilin University (Changchun, China) to improve our understanding of this disease. Written informed consent was obtained from the patient.

## Case report

The present study reports the case of a 34-year-old female (gravida 1, para 0, aborta 1) who underwent laparoscopic myomectomy for uterine fibroids at the Second Hospital of Jilin University in August 2009. During the surgery, a myoma that was 5 cm in diameter was identified in the posterior uterine wall and was removed using a morcellator. The post-operative pathology report determined that the uterus was rich in leiomyoma cells.

In May 2012, the patient underwent an exploratory laparotomy due to relapse of the uterine fibroid. During the surgery, a myoma that was 10 cm in diameter was identified at the uterine bladder peritoneal reflection, numerous unequally sized leiomyoma tubercles were identified on the uterine surface and a leiomyoma tubercle that was 2 cm in diameter was identified on the surface of the right fallopian tube. The mass was removed from the uterine bladder peritoneal reflection and frozen section analysis determined a diagnosis of uterine leiomyoma, therefore, a myomectomy was performed. Post-operative pathology determined that the lesion was a leiomyoma and immunohistochemical staining resulted in positivity for α-smooth muscle antibody (α-SMA), h-caldesmon and desmin, with a Ki-67 labeling index of 5%.

In September 2013, a gynecological ultrasound revealed a 5.0×5.0-cm mass in the left lower abdomen. As the patient presented with no specific symptoms, the patient decided to continue with follow-up examinations only. The patient was hospitalized again on 6 June, 2014, following identification of a bilateral adnexal cyst in a patient review. A gynecological ultrasound determined that the endometrium measured 0.5 cm and that the uterus and ovaries were of normal size. However, a heterogeneous hypoechoic mass was identified on each ovary, measuring 5.2×4.3 cm and 3.7×2.3 cm, respectively, and exhibiting a regular form with clear boundaries. Furthermore, color Doppler flow imaging revealed no abnormal blood flow signals and the mass activity was good under gynecological examination, demonstrating no tenderness. No abnormalities were identified in the liver, gall bladder or spleen ultrasound examinations, heart and lung assessments or gastrointestinal endoscopy. In addition, a cancer antigen 125 level of 9 U/ml (normal range, <35 U/ml), a human epididymis protein 4 level of 60.36 pM (normal range, <150 pM) and an α-fetoprotein level of 3.27 IU/ml (normal range, <10 IU/ml) were recorded.

**Figure 1:**
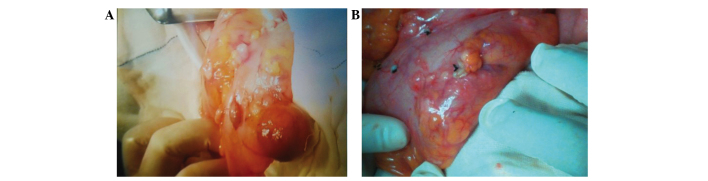
(A) Concentrated myoma tubercle-like cysts on the surface of the intestine and mesentery. (B) Following removal of the myoma tubercle-like cysts, small myoma tubercles remained, as they were too small to be removed.

**Figure 2:**
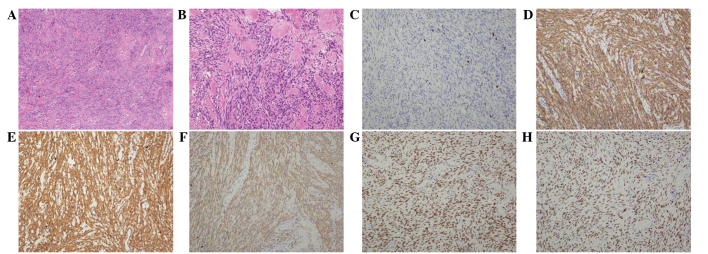
Post-operative pathological analysis to determine a diagnosis of leiomyomatosis peritonealis disseminata. Hematoxylin and eosin staining at (A) ×40 magnification and (B) ×100 magnification. Positive immunohistochemical staining for (C) Ki67 (positive rate, 1%), (D) h-caldesmon, (E) α-smooth muscle antibody, (F) desmin, (G) estrogen receptor and (H) progesterone receptor (magnification, ×100).

Based on the clinical manifestation and auxiliary examinations, ovarian neoplasm was considered as a possible diagnosis, therefore, an exploratory laparotomy was performed. During the surgery, concentrated myoma tubercle-like cysts were identified on the surface of the intestine and mesentery, with sizes ranging from 0.4×0.3×0.3 cm to 5.0×4.0×3.0 cm (Fig. 1). Additionally, a 1.5-cm diameter endometriotic cyst was identified on left ovary and a 1-cm endometriosis lesion was identified on the left posterior uterosacral ligaments. The cysts on the surface were partially removed. The frozen section indicated a diagnosis of mesenchymal tumors, however, intestinal leiomyoma could not be excluded. During the surgery, the following diagnoses were made: Intestinal leiomyoma, endometriotic cysts on the left ovary and pelvic endometriosis. The majority of the leiomyoma on the intestine and mesentery was removed, however, the myomas were compact and therefore, not all of them could be removed. The endometriotic cysts on the left ovary and the pelvic endometriosis lesion were removed concurrently. Post-operative pathology determined a diagnosis of leiomyomatosis peritonealis disseminate, with occasional mitosis and no necrosis (Fig. 2A and B). Immunohistochemical staining revealed the resected tissues to be positive for h-caldesmon, α-SMA, desmin, estrogen receptor and progesterone receptor (PR) (Fig. 2C–H), and negative for cluster of differentiation (CD)117, discovered on gastrointestinal stromal tumor-1, CD34, neuron-specific enolase and S-100, with a Ki-67 labeling index of 1%. The post-operative procedure was successful and the patient was monitored by follow-up every six months. Follow-up is scheduled for five years, after which gynecological sonography examination will be performed annually. In October 2014, a gynecological sonography examination revealed no abnormalities and at the time of writing, the patient remains alive and well.

## Discussion

In 1952, Willson and Peale (11) reported the first case of LPD. LPD predominantly occurs in females of reproductive age, however, the pathogenesis of LPD is poorly understood. The condition may be caused by changes in estrogen and progesterone levels, peritoneal metaplasia, growth factors, iatrogenic factors or genetics (12). LPD is benign, however, it exhibits recurrent and malignant tendencies. LPD lesions always appear at the omentum majus, mesentery and surface of the small intestine, or at the colon, uterus, ovary, oviduct or pouch of Douglas. LPD generally presents with no clinical symptoms, however, gastralgia or abdominal distension do occasionally occur. Previous studies, including one conducted by Larraín *et al* (9), propose that LPD may be caused by the remaining uterine fibroid fragments in the pelvic cavity, as the use of laparoscopic power morcellation during surgery could cause the fragments to be parasitized by the blood vessels of the peritoneum and mesentery (13).

In the present study, the patient was a female of reproductive age, with a surgical history of laparoscopic myomectomy for uterine fibroid, in which laparoscopic power morcellation was used. The development of myoma at the uterine bladder peritoneal reflection, leiomyoma tubercles on the uterine surface identified two years previously and multiple leiomyoma on the intestinal surface, could all be explained by the theory suggested by Larraín *et al* (9). Currently available data from an analysis conducted by the FDA (10) estimates that ~1/350 female individuals who undergo hysterectomy or myomectomy to treat fibroids also have an unsuspected uterine sarcoma. Furthermore, if these female patients with unsuspected uterine sarcomas undergo laparoscopic power morcellation during surgery, there is an increased risk of cancerous tissue dissemination within the abdomen and pelvis.

Therefore, in April 2014, the FDA published the following advice: i) Laparoscopic power morcellation during hysterectomy or myomectomy for uterine fibroids should be discouraged; ii) laparoscopic uterine power morcellation should not be performed in female patients with suspected or diagnosed uterine cancer; iii) all the possible treatment strategies should be considered for the treatment of female patients with symptomatic uterine fibroids, and the benefits and risks of each should be discussed with the patient; and iv) for those patients in whom laparoscopic power morcellation is considered to be the most appropriate therapeutic procedure, a specimen bag should be used during morcellation, with the aim of containing the uterine tissue and minimizing the risk of dissemination throughout the abdomen and pelvis (10).

Currently, there is a lack of pre-operative diagnostic methodology for LPD. Instead, intraoperative diagnosis, and intraoperative or post-operative pathology results are predominantly relied on to diagnose the condition.

In addition, no standard treatment currently exists for LPD. Certain studies have considered individualized treatments. An animal experiment demonstrated that long-term high-level progesterone administration may cause mesenchymal stem cells to develop into leiomyomatosis peritoneal lesions (14). However, a clinical study determined that estrogen stimulates subcelomic mesenchymal cells to proliferate and differentiate into myoblasts, myofibroblasts, fibroblasts and decidua-like cells (15). Therefore, the majority of studies propose that females of reproductive age should undergo lesion excision and omentectomy followed by discontinuation of hormonal disruptions (12), such as the termination of pregnancy and oral contraceptives, or the administration of oral gonadotropin-releasing hormone agonists (GnRh-a) (16), aromatase inhibitors (17) or estrogen receptor antagonists (18). A number of studies have reported that GnRh-a hormone therapy following surgery will prevent the appearance of new lesions for five years (19,20). Other studies have reported that the tumor will itself be diminished after childbirth or discontinuation of oral contraceptive (21,22), however, for young patients who desire to bear children, close follow-up visits are necessary to provide gestation guidance and to observe the change in progesterone levels.

For those patients who do not desire to bear children, total abdominal hysterectomy, salpingo-oophorectomy, omentectomy and debulking may be the most appropriate alternatives (23,24). For those patients with developing disease who cannot undergo surgery, such as PR(-) patients with liver or lung lesions, systemic chemotherapy may be undertaken (25). In the present study, the patient was a female of reproductive age who desired children. LPD was not diagnosed during surgery by frozen section, the myoma did not exhibit any uterus or adnexa involvement and concentrated myoma tubercle-like cysts were identified on the surface of the intestine; therefore, myoma resection was performed, however, the omentum, uterus and bilateral adnexa were not removed.

A British study reported that LPD is always associated with endometriosis (12); a possible explanation for this association is that the two diseases may be derived from the same cellular origin (23,25–28). During the present patient’s third surgery, an endometriotic cyst was identified on left ovary and an endometrial lesion was identified on the left posterior uterosacral ligaments. As the patient was a female of reproductive age who desired children, only the endometriotic cyst on the left ovary and the pelvic endometrial lesion were removed. The pathology results of the three surgeries undertaken by the patient were all benign, however, the disease relapsed and exhibited malignant tendencies. The pathology results of the first surgery reported abundant uterine leiomyoma cells. In the third surgery, the lesion exhibited intestinal and mesentery involvement, however, not all of the leiomyoma was removed, therefore, close follow-up examinations are required.

In conclusion, LPD is rare and difficult to diagnose, however, the prognosis is good, as the minority become malignant. The majority of LPD patients have a medical history of laparoscopic myomectomy for uterine fibroid. The use of laparoscopic power morcellation may contribute to the development of LPD, therefore, the specific surgical approach used in laparoscopic myomectomy should be carefully considered, and protective measures should be taken to prevent myoma fragments spreading if laparoscopic power morcellation is used.
